# Comparing Prescribing Behaviors and Clinician Experiences Between Multiplex PCR/Pooled Antibiotic Susceptibility Testing and Standard Urine Culture in Complicated UTI Cases

**DOI:** 10.3390/jcm13237453

**Published:** 2024-12-07

**Authors:** Emery Haley, Natalie Luke, Howard Korman, Ganesh Srinvas Rao, David Baunoch, Xiaofei Chen, Jim Havrilla, Mohit Mathur

**Affiliations:** 1 Department of Clinical Research, Pathnostics, Irvine, CA 92618, USA; ehaley@pathnostics.com (E.H.); nluke@pathnostics.com (N.L.); 2 School of Medicine, Wayne State University, Detroit, MI 48201, USA; hkorman@urologist.org; 3 Department of Urology, First Urology, Louisville, KY 40207, USA; grao@1sturology.com; 4 Department of Research and Development, Pathnostics, Irvine, CA 92618, USA; dbaunoch@pathnostics.com; 5 Department of Informatics, Pathnostics, Irvine, CA 92618, USA; xchen@pathnostics.com (X.C.); jhavrilla@pathnostics.com (J.H.); 6 Department of Medical Affairs, Pathnostics, Irvine, CA 92618, USA

**Keywords:** complicated urinary tract infection (cUTI), multiplex–polymerase chain reaction (M-PCR), pooled antibiotic susceptibility (P-AST), standard urine culture (SUC), antibiotic susceptibility, antibiotic stewardship

## Abstract

**Background/Objectives:** We aimed to compare the prescribing behavior and clinical experience of urology providers when using the combined multiplex polymerase chain reaction (M-PCR)/Pooled Antibiotic Susceptibility Testing (P-AST) diagnostic test versus the standard urine culture (SUC) in the same set of patients previously reported to have improved clinical outcomes with M-PCR/P-AST. **Methods:** We conducted a multi-centered, prospective, observational study (clinical trial registration: NCT05091931) with Western Institutional Review Board (IRB) approval (20214705). Adult subjects were split between the M-PCR/P-AST (*n* = 250) and SUC arms (*n* = 135). Treatment details were determined by clinician and subject surveys. Differences in prescribed antibiotics were compared using the Chi-square or Fisher’s exact test. **Results:** There was no significant difference in the overall use of “access” antibiotics (*p* = 1.0) or first-line drugs (*p* = 0.4483) between M-PCR/P-AST and SUC. Nitrofurantoin (*p* = 0.0172) and metronidazole (*p* = 0.0309) were more frequently used with M-PCR/P-AST, while amoxicillin/clavulanate (*p* = 0.0008), cefuroxime (*p* = 0.0378), and ertapenem (*p* = 0.0378) were more frequently used with SUC. **Conclusions:** The use of M-PCR/P-AST to guide complicated UTI management was not associated with the increased use of non-first-line antibiotics, such as carbapenems, compared to SUC. Combined with the prior reported evidence of improved patient outcomes in this same set of patients, this test should be considered for utilization when managing complicated UTI cases.

## 1. Introduction

Urinary tract infections (UTIs) account for significant healthcare costs [1] and are a major source of outpatient antibiotic prescriptions [2], especially empirically prescribed antibiotics [3]. Over the past decade, 16SrRNA sequencing and other culture-independent, molecular-based methods have revealed the existence of a resident urobiome, which includes a variety of microorganisms, including viruses, yeasts, and fastidious/anaerobic bacterial species, in addition to the previously recognized Gram-positive and Gram-negative bacterial species. [4,5,6,7,8] While the clinical relevance of these “emerging” or “opportunistic” uropathogens is still under study, the composition of the urobiome was found to change in response to aging, hormonal changes, and antibiotic use, suggesting a role for urobiome dysbiosis in UTI risk and pathogenesis [9,10,11,12]. Critically, in contrast to molecular methods, SUC has been repeatedly demonstrated to lack sensitivity for detecting most of these organisms [13,14,15,16]. The culture conditions of SUC favor the growth of Gram-negative bacteria, especially *Escherichia coli (E. coli)*, which is the most commonly identified organism in acute UTIs [17]. Yet, “emerging” or “opportunistic” uropathogens found in the urobiome have been demonstrated to contribute to clinically significant lower urinary tract symptoms [18]. Consistent with this limitation in sensitivity, a recent publication has shown poor outcomes for patients with negative SUC results but whose urine specimens contained viable microorganisms, which grew under more sensitive expanded culture conditions [19]. In this study, many SUC-positive cases also experienced poor outcomes. This result may be due to heteroresistance [20,21,22,23,24,25,26,27,28,29,30,31], limitations in the recognition of polymicrobial infections, and/or the inability to assess full antibiotic susceptibility within polymicrobial infections [32]. Recent research in the field has demonstrated that polymicrobial infections are common, particularly in older adults and complicated UTIs [32,33,34,35,36,37,38]. Also, SUC followed by a standard antibiotic susceptibility test (AST) frequently requires ≥3 days to return the results, which is longer than many clinicians and patients are willing to delay treatment; a standard AST typically measures the susceptibility using a few isolates, and therefore, may fail to capture heteroresistant infections [39]. Advanced diagnostic tests are now available that seek to address these limitations of SUC.

One such test is Guidance^®^ UTI, a combined multiplex polymerase chain reaction (M-PCR) and Pooled Antibiotic Susceptibility Testing (P-AST) assay, which addresses both the limited sensitivity of SUC and the inability of M-PCR alone to provide phenotypic antibiotic susceptibility results. The M-PCR component provides pathogen identification and the detection of resistance genes and has shown improved sensitivity and high specificity compared to SUC [33,35,40,41,42]. Positive M-PCR results also exhibit high concordance with the identification of viable organisms grown by the expanded culture methodology [19], indicating that M-PCR is capable of detecting viable organisms. It is important to note that while the organisms targeted by the M-PCR assay have all been associated with UTI pathogenesis, their detection by M-PCR or SUC does not inherently indicate pathogenicity. In a prior study of cases that were clinically assessed as having a presumptive UTI, organisms detected by M-PCR were highly associated with elevated inflammatory markers of the urinary tract [40,41,42]. This indicates that there is a high correlation between organism detection and pathogenicity in the appropriate patient population and highlights the importance of assessing UTI symptoms and the clinical workup when utilizing diagnostic tests.

Additionally, M-PCR produces quantitative results (in cells/mL) that correlate linearly 1:1 with the CFU/mL units used in SUC [43]. The P-AST component tests for pooled organism growth in Mueller–Hinton broth against multiple dilutions of UTI-relevant antibiotics using a fluorescent probe. This accounts for the effects of multi-species interactions that may alter susceptibility in polymicrobial infections and for the presence of multiple strains of a single species, which can co-occur in a UTI with varying susceptibilities (heteroresistance) [44,45,46]. The M-PCR/P-AST assay has prior publications demonstrating that (1) the clinical use of this test is associated with improved patient outcomes and (2) the test is rapid, typically completed within one day of the specimen’s receipt at the testing lab [32,47,48,49].

When assessing the utility of these new diagnostic tests compared to the culture, an important consideration beyond improvements in patient outcomes is whether first-line antibiotics are prioritized before resorting to antibiotics reserved for non-responsive and multi-drug-resistant infections. In 2017, the World Health Organization (WHO) developed the Access, Watch, and Reserve (AWaRe) classification system of antibiotics to aid in global antimicrobial stewardship efforts [50,51], AWaRe classifies antibiotics according to both their spectrum of activity and their potential to contribute to increasing antimicrobial resistance. The “access” classification applies to antibiotics used in first-line treatment with a low risk of developing resistance. The “watch” classification applies to antibiotics with a broader spectrum of activity and a higher risk of developing resistance. According to the WHO, nitrofurantoin is the preferred treatment option for acute lower UTIs, and amoxicillin/clavulanate, trimethoprim, or sulfamethoxazole/trimethoprim are additional first-line “access” antibiotic options [51].

The Infectious Diseases Society of America (IDSA) and the European Society for Microbiology and Infectious Disease joint guidelines also recommend nitrofurantoin or sulfamethoxazole/trimethoprim for a first-line lower UTI treatment [52]. They also include fosfomycin as a first-line treatment in contrast to the WHO classification of fosfomycin as a “watch” antibiotic [51,52]. Due to high resistance rates in a growing number of locations, sulfamethoxazole/trimethoprim is recommended as a frontline treatment for acute lower UTIs only when current local antibiograms indicate that resistance rates are <20% or when an individual patient’s specimen is tested and found susceptible, which makes it less useful as a form of empiric therapy [52]. Similarly to IDSA, the American Urological Association’s (AUA’s) guidelines for recurrent UTIs recommend nitrofurantoin, sulfamethoxazole/trimethoprim, and fosfomycin as first-line antibiotics, with the caveat that sulfamethoxazole/trimethoprim should not be used where local resistance exceeds 20%.

The M-PCR/P-AST assay studied here has previously demonstrated an improvement in patient outcomes and a reduction in empiric therapy use [47]. The objective of this study is to analyze those exact same cases and compare the use of first-line antibiotics and provider experience when using M-PCR/P-AST compared to SUC.

## 2. Materials and Methods

### 2.1. Study Design and Methodology

This study was a multi-centered, prospective, observational study (clinical trial registration: NCT05091931, https://clinicaltrials.gov/ct2/show/NCT05091931 (accessed on 20 September 2024). It was conducted in accordance with the Declaration of Helsinki and with the approval of the Western Institutional Review Board—Copernicus Group (WGC-IRB) (ID: 20214705), who determined that the study protocol met all requirements for a partial waiver of authorization and required only that verbal informed consent was given prior to enrollment by all subjects.

As an observational study that aimed to compare patient outcomes and provider prescribing behavior when either MCR/P-AST or SUC are utilized, the test cohort assignment was at the clinicians’ discretion, and only the assigned test result was provided. Additionally, the clinicians were free to treat the patient (whether based on test results, clinical experience, expertise, or both) at their discretion without direction from the trial protocol. This study was designed to determine if any significant differences exist between test cohorts in patient outcomes, including the choice of whether to prescribe any antibiotic treatment, whether to prescribe empiric versus directed antibiotic treatment and which antibiotic treatments were prescribed empirically vs. which antibiotic treatments were prescribed as directed therapy. The study included a broad patient population representing a typical clinical caseload for a urology office.

The cohort consisted of 385 adult (≥18 years old) subjects (*n* = 112 males and *n* = 273 females) who presented to urology or urogynecology clinics with symptoms and clinical presentations consistent with complicated UTIs from 2 U.S. states between 30 March 2022 and 24 May 2023, who required microbial diagnostic testing according to their clinician’s judgment, and who were willing and able to provide informed consent in English or Spanish. Individuals who were pregnant, incarcerated, currently taking antibiotics, currently receiving radiotherapy, relying on self-catheterization or a chronic indwelling catheter, diagnosed with bladder/urologic cancer, chronic pelvic pain, untreated overactive bladder, or who had urinary diversion were excluded. The full inclusion and exclusion criteria, including the definition of cUTI, are described in the prior publication of this study dataset [47]. The subjects entered either the M-PCR/P-AST (*n* = 250) or the SUC (*n* = 135) arm based on the test requisition order at their clinician’s discretion. The healthcare provider documented the subjects’ clinical presentations, medical history, and treatment plans. The subjects provided midstream voided or in-and-out catheter-collected urine specimens and completed baseline and daily surveys [47].

Treatment status was determined, as previously described [47], based on both the clinical evaluation forms completed by healthcare providers and on the subjects’ responses in daily surveys [47]. “Treated” was defined as treated with antimicrobials, including antibiotics and anti-fungal medications, between day 1 and day 14 of the study. Subjects who either did not receive any medication(s) at all or who only received medication other than antimicrobials (ex: pain medication) were defined as “untreated”.

Treated subjects were categorized as receiving “empiric treatment” if the healthcare provider or subjects reported empirical treatment via the questions on the forms/surveys. If the form did not have this information, antibiotics reported as being given prior to the test result being provided were considered empiric. Treated subjects were categorized as receiving “directed treatment” if the healthcare provider or subjects reported the use of directed treatment via the questions on the forms/surveys. If the form did not have this information, antibiotics given after the test result was provided were considered as receiving “directed treatment”. Subjects who received antimicrobial treatment both before and after the test results were made available were categorized as receiving both empiric and directed treatment, whether the provider continued the same antibiotic or changed it.

### 2.2. Standard Urine Culture and Antibiotic Susceptibility Testing

In the case that SUC was selected, the clinician submitted a test requisition per their usual clinical practices. These tests were performed by separate clinical diagnostic labs with high-complexity CLIA certifications that were already routinely being used by the providers/medical office prior to the study, following the standard protocols of each laboratory. These labs are independent of the authors, and no changes were requested to their standard operation procedures for this study. This setup best provides an unbiased SUC comparator that mirrors general clinical lab results for SUC at multiple sites.

### 2.3. M-PCR/P-AST Assay (Guidance^®^ UTI, Offered by Pathnostics, Irvine, CA, USA)

As described previously [33,47], DNA extracted from the subject’s urine sample using a King Fisher/MagMAX™ automated DNA extraction instrument and the MagMAX™ DNA Multi-Sample Ultra Kit (Thermo Fisher, Carlsbad, CA, USA) was mixed with a universal PCR master mix and amplified using TaqMan technology in a Life Technologies 12K Flex 112-format Open Array System (Thermo Fisher Scientific, Wilmington, NC, USA). For the M-PCR component of the assay, probes, and primers are used to detect 32 antibiotic-resistance genes as well as the following microorganisms, which included 4 yeast species, and 26 bacterial species or groups, both fastidious and non-fastidious:

*Candida albicans (C. albicans), Candida glabrata (C. glabrata), Candida parapsilosis (C. parapsilosis), Citrobacter freundii (C. freundii), Citrobacter koseri (C. koseri), Enterococcus faecalis (E. faecalis), Enterococcus faecium (E. faecium), Escherichia coli (E. coli), Klebsiella oxytoca (K. oxytoca), Klebsiella pneumoniae (K. pneumoniae), Morganella morganii (M. morganii), Pantoea agglomerans (P. agglomerans), Proteus mirabilis (P. mirabilis), Providencia stuartii (P. stuartii), Pseudomonas aeruginosa (P. aeruginosa), Serratia marcescens (S. marcescens), Staphylococcus aureus (S. aureus), Streptococcus agalactiae (S. agalactiae), Enterobacter* group [including *Klebsiella aerogenes* (formally known as *Enterobacter aerogenes*) and *Enterobacter cloacae*], *Acinetobacter baumannii (A. baumannii), Actinotignum schaalii (A. schaalii), Aerococcus urinae (A. urinae), Alloscardovia omnicolens (A. omnicolens), Candida auris (C. auris), Corynebacterium riegelii (C. riegelii), Gardnerella vaginalis (G. vaginalis), Mycoplasma hominis (M. hominis), Ureaplasma urealyticum (U. urealyticum)*, coagulase-negative *staphylococci* group (CoNS) (including *Staphylococcus epidermidis*, *Staphylococcus haemolyticus*, *Staphylococcus lugdunesis*, and *Staphylococcus saprophyticus*), and Viridans group *streptococci* (VGS) (including *Streptococcus anginosus, Streptococcus oralis*, and *Streptococcus pasteuranus*).

In addition, the fluorescence-based P-AST was performed on all specimens in which the M-PCR test component identified at least one non-fastidious bacterial species or group capable of growth in the assay conditions. The method was performed, as described previously [47] to determine susceptibility to the following 19 antibiotics commonly used for UTI treatment: amoxicillin/clavulanate, ampicillin, ampicillin/sulbactam, cefaclor, cefazolin, cefepime, cefoxitin, ceftazidime, ceftriaxone, ciprofloxacin, fosfomycin, gentamicin, levofloxacin, meropenem, nitrofurantoin, piperacillin/tazobactam, sulfamethoxazole, tetracycline, and vancomycin.

### 2.4. Statistical Analysis

**Figure 1:**
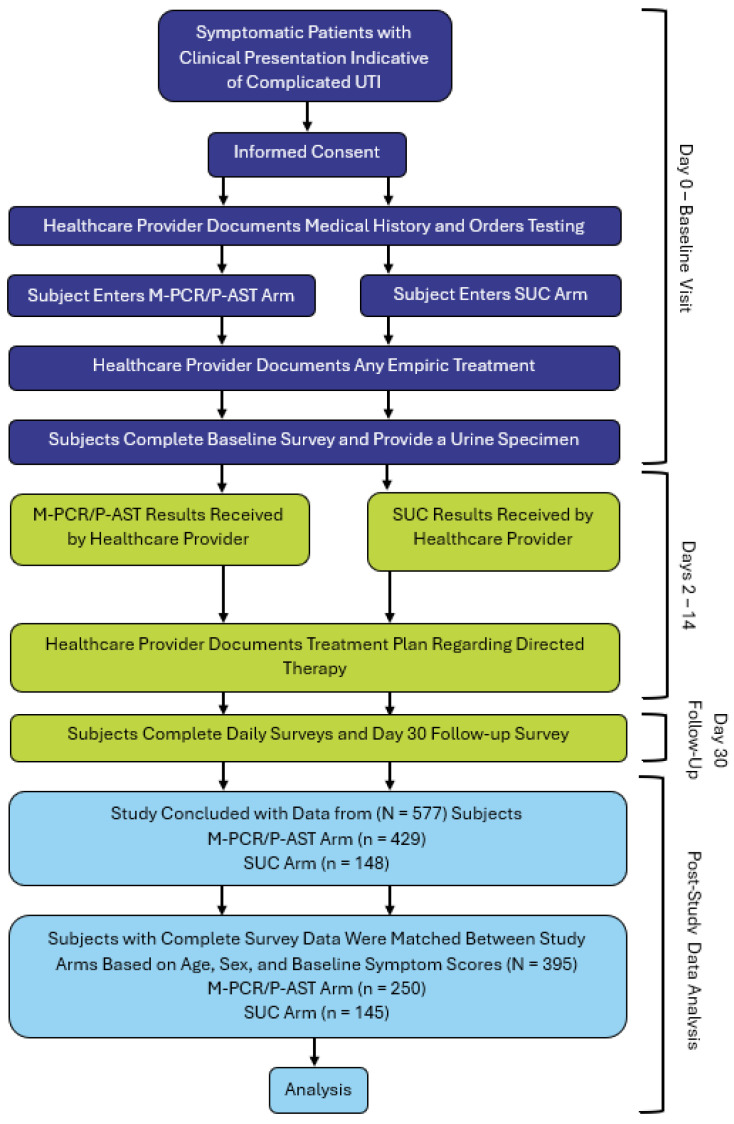
Study events and subject selection for analysis.

To reduce bias, subjects were matched between the two arms on categories of age, sex, and baseline United States Food and Drug Administration (FDA) symptom scores, as described in the 2023 publication by Haley et al. [47], prior to analysis. A total of 385 subjects were matched between the M-PCR and SUC cohorts for this analysis. The Chi-squared test or Fisher’s exact test was used to test whether the difference between the SUC and M-PCR/PAST cohorts was significant. For all statistical tests, significance was defined as *p* < 0.05. No adjustments were made for multiple comparisons. All statistical calculations were performed using Python 3.10.12 (Figure 1).

## 3. Results

### 3.1. Subject Demographics

A total of 395 individuals were successfully matched between the M-PCR/P-AST (*n* = 250) and SUC (*n* = 145) arms based on sex, age, and baseline symptom scores (Table 1). The matched cases were approximately 70% female (*n* = 176/250 for M-PCR and *n* = 103/145 for SUC) with no statistical difference in sex distribution between the cohorts (*p* = 0.4931). The resulting mean age was approximately 65 years and was similar between the M-PCR and SUC cohorts (*p* = 0.9093). Baseline symptom scores between the two arms were also similar after matching (*p* = 0.5306). Details of organisms detected and the number of polymicrobial cases are provided in Supplemental Tables S1 and S2.

### 3.2. Treatment Decisions

There were no significant differences between the cohorts for the proportion of treated versus untreated subjects (*p* = 0.0548) (Table 2a). However, there was a significant difference in the distribution of empiric-only, mixed, or directed-only treatments (*p* = 0.0034), with the higher utilization of empiric therapy in the SUC arm (Table 2b).

For empiric therapy, only amoxicillin/clavulanate had a statistically significant difference (*p* = 0.0309) with increased use in the SUC cohort compared to M-PCR/P-AST (Supplemental Table S3).

Comparing directed therapies, nitrofurantoin (*p* = 0.0172) and metronidazole (*p* = 0.0309) were used more in the M-PCR/P-AST cohort, while amoxicillin/clavulanate (*p* = 0.0008), cefuroxime (*p* = 0.0378), and ertapenem (*p* = 0.0378) were used more in the SUC cohort (Table 3). Metronidazole was used in the M-PCR/P-AST arm for nine cases where *G. vaginalis* was detected, which is not detectable by SUC.

There was no significant difference between the cohorts for the utilization of “watch” classification antibiotics versus “access” classification antibiotics selected for directed treatment (*p* = 1.0) (Table 4a).

There was also no significant difference between the cohorts in the utilization of “first-line” classification antibiotics, fosfomycin, nitrofurantoin, and sulfamethoxazole/trimethoprim, during the directed treatment (*p* = 0.4483) (Table 4b).

The results of a survey for the providers in this study show the use and importance of each component of the M-PCR/P-AST assay (Supplemental Figure S1), with 71% of providers indicating that all components together were important. For those who ranked the individual components of the M-PCR/P-AST results in order of importance, the P-AST result was ranked the most important (47% gave P-AST the highest rank).

## 4. Discussion

There is a major gap in the efficacy of clinical care for complicated and recurrent UTIs due to the significant limitations of the current standard of care diagnostic tests, leading to high rates of hospitalization and ER/Urgent Care visits [47]. Equally important, these patients have a significantly reduced quality of life due to the persistence and recurrence of symptoms, including pain [53,54,55,56,57,58,59,60,61,62,63]. Prior published studies have already demonstrated that the M-PCR/P-AST diagnostic test is associated with reduced hospitalizations, ER visits, Urgent Care visits, and the recurrence of UTI symptoms [47,48]. Unlike M-PCR tests alone, the P-AST component of the test provides antibiotic susceptibility information, thereby providing phenotypic susceptibility results to the clinician. Urology providers indicated that these results are important to them as they make prescribing and patient management decisions (Supplemental Figure S1), with both the M-PCR and P-AST components being selected as important to decision-making.

The primary aim of this report was to assess whether the utilization of the novel M-PCR/P-AST diagnostic assay resulted in any change to the use of first-line or “access” antibiotics versus the standard-of-care diagnostic test, SUC, for patients with a suspected complicated UTI. Secondarily, the total number of antibiotic prescriptions, the category of prescription timing (empiric or test-result-directed), and the specific antibiotics chosen were analyzed. Prior reports have assessed the impact on patient outcomes of using this advanced diagnostic, showing an improvement compared to SUC and a significant reduction in empiric therapy in the same set of patients studied here [47].

Overall, the results show there was no significant difference in the use of WHO-classified “watch” antibiotics nor “access” antibiotics between the M-PCR/P-AST assay and standard urine culture cohorts (*p* = 1.0). Both tests also had no significant differences in their use of the first-line therapies listed by the Infectious Diseases Society of America (IDSA) and the American Urological Association (AUA) (*p* = 0.4483). This demonstrates that there was no significant association between the utilization of the Guidance UTI test (M-PCR/P-AST) and increased use of non-first-line antibiotics. The results do not imply equivalency between the antibiotics used in the two arms but do show that the improved outcomes shown previously for the M-PCR/P-AST arm were not associated with the increased utilization of non-first-line antibiotics.

Additionally, comparing the antibiotics chosen for empiric therapy, there was only one statistically significant difference between the SUC and M-PCR/P-AST cohorts, specifically, that amoxicillin/clavulanate was used more in the SUC cohort (*p* = 0.0309). Given that the empiric selection of an antibiotic is made based on clinical judgment and local antibiograms rather than on actual test results, the lack of significant difference for almost all antibiotics is expected.

There was a slightly larger number of antibiotic prescriptions in the M-PCR/P-AST cohort, which did not reach statistical significance between the cohorts in the proportion of treated versus untreated subjects (*p* = 0.0548). There was a significantly higher utilization of empiric therapy in the SUC arm compared to M-PCR/P-AST (*p* = 0.0034). These results are consistent with our previous data, which also showed a reduction in empiric therapy with the utilization of M-PCR/P-AST [47,48].

For directed therapies, nitrofurantoin (*p* = 0.0172) was used more in the M-PCR/P-AST cohort than with SUC. Nitrofurantoin is widely recommended as a first-line treatment for UTIs [51,52], and increased utilization is consistent with good antimicrobial stewardship. Metronidazole was used as directed therapy for nine cases in the M-PCR/P-AST cohort but not at all in the SUC cohort, presumably due to the inability of SUC to detect *G. vaginalis*. The M-PCR/P-AST assay adds a comment that metronidazole, along with other relevant antimicrobials, are commonly used for management when *G. vaginalis* is detected, though it does not run pooled susceptibility (P-AST) against metronidazole. *G. vaginalis* is a fastidious, anaerobic bacteria of the urogenital microbiome [64,65,66,67,68,69,70,71], historically considered a contaminant in urine [69,70,72,73]. However, recent data have shown that in the bladder, *G. vaginalis* is associated with immune activation, urothelial exfoliation, recurrent UTIs, and urosepsis [74,75,76,77,78]. Metronidazole has demonstrated efficacy in treating *G. vaginalis* UTIs [79].

In the SUC cohort, amoxicillin/clavulanate (*p* = 0.0008) and cefuroxime (*p* = 0.0378) were used more for directed treatment than in the M-PCR/P-AST cohort. Amoxicillin/clavulanate is a beta-lactamase inhibitor combination class “access” antibiotic recommended by the WHO for frontline lower UTI treatment [51]. However, this combination is not a first-line treatment according to the IDSA and the European Society for Microbiology and Infectious Disease joint guidelines [52] and is strongly associated with gastrointestinal side effects, including *Clostridium difficile* infection [80]. Cefuroxime, a cephalosporin antibiotic, is a “watch” classification antibiotics whose use should be minimized [51].

Ertapenem, as a directed therapy, was used exclusively in the SUC cohort with no use in the M-PCR/P-AST cohort (*p* = 0.0378), though the number of cases where it was used was small (*n* = 3 in the SUC arm). This does not imply that ertapenem usage was inappropriate in the SUC arm since the presence of ESBL or other factors can make its use necessary. It does indicate that M-PCR/P-AST is not associated with an increase in the use of this non-first-line carbapenem class antibiotic.

### Limitations and Future Directions

The focus of this study was on clinician-prescribing behavior to assess if there were any changes to the use of first-line and “access” antibiotics with the use of the new diagnostic, using the same dataset of patients that demonstrated improved outcomes in a prior report [47]. Future studies may assess more detailed criteria by which clinicians can arrive at their treatment decisions and examine case-by-case correlations between which organism(s) are present, what the antibiotic susceptibility test results were, which specific antibiotic(s) have been prescribed, and the patient’s resulting clinical outcome(s). This study also does not imply causation between the use of first-line therapy and the improved outcomes reported in prior work, nor does it imply equivalency with SUC antibiotic choices, which may also be a focus of further research. As a novel diagnostic, further study of the Pooled Antibiotic Susceptibility (P-AST) component, including a comparison to standard methods of measuring isolates’ antibiotic susceptibility, would be useful and are currently ongoing. These studies will also standardize the SUC assay at a central laboratory instead of the real-world method used here, where SUC testing was performed at multiple independent laboratories. A larger study may also be considered to further assess outcomes such as overall antibiotic usage rates and those outlined above.

## 5. Conclusions

This study demonstrated that the use of the M-PCR/P-AST assay to guide complicated UTI diagnosis and treatment did not significantly change the proportion of first-line or “access” antibiotics used, nor the overall proportion of treatment versus non-treatment, compared to standard urine culture. The previous finding that M-PCR/P-AST was associated with the reduced utilization of empiric antibiotic therapy was also replicated here. This study, combined with previous studies on the same set of patients, demonstrates that the M-PCR/P-AST assay has the potential to improve patient outcomes in complicated and recurrent UTI cases without increasing the use of non-first-line antibiotics. The use of this novel diagnostic should be part of a broader clinical assessment, and management should be based on clinical judgment, including the consideration of our current understanding of the urinary microbiome.

## Figures and Tables

**Table 1:** Demographics of the matched study cohorts.

	All Subjects (*n* = 395)		
	SUC (*n* = 145)	M-PCR/P-AST (*n* = 250)	*p*-Value
Age			0.4931
Mean (SD)	63.0 (14.8)	64.0 (13.7)	
Median (Min, Max)	65.8 (21.0, 95.7)	66.5 (22.0, 96.1)	
Sex [*n* (%)]			0.9093
Male	42 (29.0%)	74 (29.6%)	
Female	103 (71.0%)	176 (70.4%)	
Mean Baseline Symptom Score (SD)	6.5 (3.5)	6.3 (3.2)	0.5306

**Table 2:** (**a**) Prevalence of antimicrobial treatment between SUC and M-PCR/P-AST cohorts. (**b**) Comparing proportions of empiric versus directed treatments between SUC and M-PCR/P-AST cohorts.

**(a)**					
**Total (*n* = 395)**	**SUC**		**M-PCR/P-AST**		***p*-Value**
	**(*n* = 145)**		**(*n* = 250)**		
	***n***	***%***	***n***	***%***	0.0548
Not Treated with Antimicrobial Agents	45	*31.0*	55	*22.0*	
Treated with Antimicrobial Agents	100	*69.0*	195	*78.0*	
**(b)**					
**Total (*n* = 295)**	**SUC**		**M-PCR/P-AST**		***p*-Value**
	**(*n* = 100)**		**(*n* = 195)**		
	***n***	**%**	***n***	**%**	0.0034
Empiric Only	2	2.0	4	2.1	
Empiric to Directed	71	71.0	99	50.8	
Directed Only	27	27.0	92	47.2	

**Table 3:** Antibiotic selections for directed treatments between SUC and M-PCR/P-AST cohorts.

2019 WHO AWaRe Classification	Antibiotic Class	Antibiotic Name	Directed Treatment				*p*-Value
			SUC (*n* = 96)		M-PCR/P-AST (*n* = 188)		
			*n*	%	*n*	%	
Access	Nitrofuran	Nitrofurantoin	18	18.8%	61	32.4%	**0.0172**
Watch	Phosphonic	Fosfomycin	3	3.1%	6	3.2%	1.0000
Access	Sulfonamide/ Dihydrofolate Reductase Inhibitor	Sulfamethoxazole/ Trimethoprim	17	17.7%	17	9.0%	0.0517
Access	Dihydrofolate Reductase Inhibitor	Trimethoprim *	1	1.0%	0	0.0%	0.3380
Access	Penicillin	Amoxicillin *	0	0.0%	1	0.5%	1.0000
Access		Ampicillin	1	1.0%	8	4.3%	0.2810
Access	Beta-lactamase Inhibitor Combination	Amoxicillin/ Clavulanate	17	17.7%	9	4.8%	**0.0008**
Watch	Cephalosporins	Ceftriaxone	0	0.0%	0	0.0%	1.0000
Watch		Cefdinir *	1	1.0%	1	0.5%	1.0000
Access		Cefuroxime *	3	3.1%	0	0.0%	**0.0378**
Access		Cephalexin *	2	2.1%	8	4.3%	0.5032
Watch	Fluoroquinolones	Levofloxacin	9	9.4%	27	14.4%	0.2629
Watch		Ciprofloxacin	14	14.6%	24	12.8%	0.7138
Watch	Carbapenems	Ertapenem *	3	3.1%	0	0.0%	**0.0378**
Access	Tetracyclines	Doxycycline *	7	7.3%	17	9.0%	0.8219
Access	Nitroimidazoles	Metronidazole	0	0.0%	9	4.8%	**0.0309**

Antibiotics marked with an * were not tested by P-AST. Bolded *p*-values indicate statistical significance.

**Table 4:** (**a**) Comparison of utilization of watch versus access antibiotics for directed treatments between SUC and M-PCR/P-AST cohorts. (**b**) Comparison of utilization of first-line antibiotics (nitrofurantoin, fosfomycin, and sulfamethoxazole/trimethoprim) for directed treatments between SUC and M-PCR/P-AST cohorts.

(**a**)					
**All Directed Treatments (*n* = 284)**	**SUC (*n* = 96)**		**M-PCR/P-AST (*n* = 188)**		***p*-Value**
	***n***	**%**	***n***	**%**	
Watch Antibiotics (*n* = 88)	30	31.2	58	30.9	1.0
Access Antibiotics (*n* = 196)	66	68.8	130	69.1	
(**b**)					
**All Directed Treatments (*n* = 284)**	**SUC (*n* = 96)**		**M-PCR/P-AST (*n* = 188)**		***p*-Value**
	***n***	**%**	***n***	**%**	
First-Line Antibiotics (*n* = 122)	38	39.6	84	44.7	0.4483
Other Antibiotics (*n* = 162)	58	60.4	104	55.3	

## Data Availability

The original data presented in the study are openly available in FigShare at DOI: 10.6084/m9.figshare.27324660.

## Supplementary Materials

The following supporting information can be downloaded at https://www.mdpi.com/article/10.3390/jcm13237453/s1. Supplemental Table S1: The proportion of monomicrobial versus polymicrobial infections detected by M-PCR/P-AST compared to SUC; Supplemental Table S2: Histogram of detected organisms; Supplemental Table S3: Antibiotic selections for empiric treatments between SUC and M-PCR/P-AST cohorts; Table S4. Survey Questions and Response Options; Supplemental Figure S1: Results of survey ranking importance of report components.
