# Supplementary material for: Comparing Prescribing Behaviors and Clinician Experiences Between Multiplex PCR/Pooled Antibiotic Susceptibility Testing and Standard Urine Culture in Complicated UTI Cases
Source: J Clin Med. 2024 Dec 7;13(23):7453. doi: 10.3390/jcm13237453 (PMC11642584; doi:10.3390/jcm13237453)

Supplemental Table S1. The proportion of Monomicrobial versus Polymicrobial Infections Detected by M-PCR/P-AST Compared to SUC

Organism	Collection Method	# of positive cases	% of positive cases	p value
**Overall**	*M-PCR/P-AST*	179	*91.8%*	**<0.0001**
	SUC	56	*56.0%*	
Polymicrobial	*M-PCR/P-AST*	119	*61.0%*	**<0.0001**
	SUC	8	*8.0%*	
Monomicrobial	*M-PCR/P-AST*	60	*30.8%*	0.0049
	SUC	48	*48.0%*	

Supplemental Table S2. Histogram of Detected Organisms

*“Other” organisms included in SUC results, but not reported by M-PCR/P-AST included: “mixed gram-positive organisms” (n = 1), “alpha-hemolytic *Streptococcus* species” (n = 2), “beta-hemolytic *Streptococcus* species” (n = 2), unspecified “*Enterococcus* species” (n = 2), unspecified species of “gram-negative bacilli” (n = 1), and *Proteus penneri* (n = 1).

Group	Organism	Collection Method	# of positive cases	% of positive cases	p value
CLASSICAL	*E. coli*	*M-PCR/P-AST*	81	*41.5%*	0.9009
		SUC	43	*43.0%*	
	E. faecalis	*M-PCR/P-AST*	37	*19.0%*	**<0.0001**
		SUC	2	*2.0%*	
	K. pneumoniae	*M-PCR/P-AST*	12	*6.2%*	**0.0180**
		SUC	15	*15.0%*	
	P. mirabilis	*M-PCR/P-AST*	7	*3.6%*	0.0610
		SUC	9	*9.0%*	
	Enterobacter Group	*M-PCR/P-AST*	6	*3.1%*	0.4294
		SUC	1	*1.0%*	
	P. auruginosa	*M-PCR/P-AST*	6	*3.1%*	0.0995
		SUC	0	*0.0%*	
	C. koseri	*M-PCR/P-AST*	4	*2.1%*	0.3036
		SUC	0	*0.0%*	
	K. oxytoca	*M-PCR/P-AST*	4	*2.1%*	0.6653
		SUC	1	*1.0%*	
	M. morganii	*M-PCR/P-AST*	3	*1.5%*	1.0000
		SUC	1	*1.0%*	
	E. faecium	*M-PCR/P-AST*	3	*1.5%*	0.5534
		SUC	0	*0.0%*	
	S. aureus	*M-PCR/P-AST*	3	*1.5%*	0.5534
		SUC	0	*0.0%*	
	S. marcescens	*M-PCR/P-AST*	3	*1.5%*	0.5534
		SUC	0	*0.0%*	
	C. freundii	*M-PCR/P-AST*	2	*1.0%*	0.5503
		SUC	0	*0.0%*	
EMERGING	*A. schaalii*	*M-PCR/P-AST*	58	*29.7%*	**<0.0001**
		SUC	0	*0.0%*	
	VGS	*M-PCR/P-AST*	59	*30.3%*	**<0.0001**
		SUC	0	*0.0%*	
	A. urinae	*M-PCR/P-AST*	57	*29.2%*	**<0.0001**
		SUC	0	*0.0%*	
	G. vaginalis	*M-PCR/P-AST*	21	*10.8%*	**0.0002**
		SUC	0	*0.0%*	
	CoNS	*M-PCR/P-AST*	14	*7.2%*	0.0999
		SUC	2	*2.0%*	
	S. agalactiae	*M-PCR/P-AST*	12	*6.2%*	**0.0099**
		SUC	0	*0.0%*	
	C. riegelii	*M-PCR/P-AST*	8	*4.1%*	0.0547
		SUC	0	*0.0%*	
	A. omnicolens	*M-PCR/P-AST*	10	*5.1%*	**0.0180**
		SUC	0	*0.0%*	
	U. urealyticum	*M-PCR/P-AST*	12	*6.2%*	**0.0099**
		SUC	0	*0.0%*	
	M. hominis	*M-PCR/P-AST*	3	*1.5%*	0.5534
		SUC	0	*0.0%*	
	A. baumannii	*M-PCR/P-AST*	1	*0.5%*	1.0000
		SUC	0	*0.0%*	
YEAST	*C. glabrata*	*M-PCR/P-AST*	5	*2.6%*	0.1707
		SUC	0	*0.0%*	
	C. albicans	*M-PCR/P-AST*	3	*1.5%*	0.5534
		SUC	0	*0.0%*	
	"Other"	*SUC*	9	*9.0%*	NA

Supplemental Table S3. Antibiotic Selections for Empiric Treatments Between SUC and M-PCR/P-AST Cohorts

2019 WHO AWaRe Classification	Antibiotic Class	Antibiotic Name	Empiric Treatment SUC (n = 79) n		M-PCR/P-AST (n = 108) n		p-value
			n	%	n	%	
Access	**Nitrofurans**	Nitrofurantoin	18	*22.8*	29	*26.9*	0.6096
Watch	**Phosphonics**	Fosfomycin	4	*5.1*	1	*0.9*	0.1641
Access	**Sulfonamide/ Dihydrofolate Reductase Inhibitors**	Sulfamethoxazole/ Trimethoprim	13	*16.5*	20	*18.5*	0.8464
Access	**Dihydrofolate Reductase Inhibitors**	Trimethoprim*	1	*1.3*	0	*0*	0.4225
Access	**Penicillins**	Amoxicillin*	0	*0*	0	*0*	1.0000
Access		Ampicillin	0	*0*	1	*0.9*	1.0000
Access	**Beta-lactamase Inhibitor Combinations**	Amoxicillin/Clavulanate	9	*11.4*	3	*2.8*	**0.0306**
Watch	**Cephalosporins**	Ceftriaxone	0	*0*	1	*0.9*	1.0000
Access		Cephalexin*	6	*7.6*	15	*13.9*	0.2419
Watch		Cefuroxime	0	*0*	0	*0*	1.0000
Watch		Cefdinir*	2	*2.5*	0	*0*	0.1772
Watch	**Fluoroquinolones**	Levofloxacin	8	*10.1*	8	*7.4*	0.5998
Watch		Ciprofloxacin	10	*12.7*	15	*13.9*	0.8320
Access	**Tetracyclines**	Doxycycline	7	*8.9*	14	*13.0*	0.4841
Access	**Nitroimidazoles**	Metronidazole	0	*0*	1	*0.9*	1.0000
Watch	**Carbapenems**	Ertapenem	1	*1.3*	0	*0*	0.4225

*denotes an antibiotic NOT included on the M-PCR/P-AST Result Report

Supplemental Table S4. Survey Questions and Response Options

Question	Responses
1) Do you consider all components of the Guidance® UTI report (labeled A-D on the sample report image) to be equally important for your decision-making process when prescribing antibiotics for complex, recurrent, or persistent UTIs, or are some components more impactful than others?<br>If equal, fill in "yes" below; if not, fill in "no," and you will be directed to a question where you can rank the components.	Yes, I consider all components of the Guidance® UTI report to be equally important [Jump to Question 3]
	No, some components are more impactful than other [Proceed to Question 2]
2) Please rank the following components of the Guidance® UTI result report in order of importance for your decision-making process when prescribing antibiotics for complex, recurrent, or persistent UTIs.<br>(#1/top is most important and #4/bottom is least important)	Rank (Note that options were presented in randomized order):<br>A. Pooled Antibiotic Susceptibility (P-AST)<br>B. Organism Identity and Microbial Density<br>C. Antibiotic Efficacy Chart<br>D. Presence/Absence of Antibiotic Resistance Genes<br>[See Figure 2 for a sample report with labeled components]
3) Specifically, does the Pooled Antibiotic Susceptibility Testing (P-AST) component of Guidance® UTI impact your decisions on which antibiotic to prescribe for your recurrent, persistent, or complicated UTI cases?	Yes
	No
4) How often do you rely on the results from pooled antibiotic susceptibility testing (P-AST) when prescribing antibiotics for patients with complex, recurrent, or persistent urinary tract infections (UTIs)?	Always
	Usually
	Sometimes
	Rarely
	Never
5) In your experience, how effectively does pooled antibiotic susceptibility testing (P-AST) in the Guidance® UTI assay help manage patients with complex, recurrent, or persistent urinary tract infections?	Extremely Effective
	Very Effective
	Somewhat Effective
	Not So Much/Not At All Effective

Supplemental Figure S1. Results of Question Two - Ranking Report Components

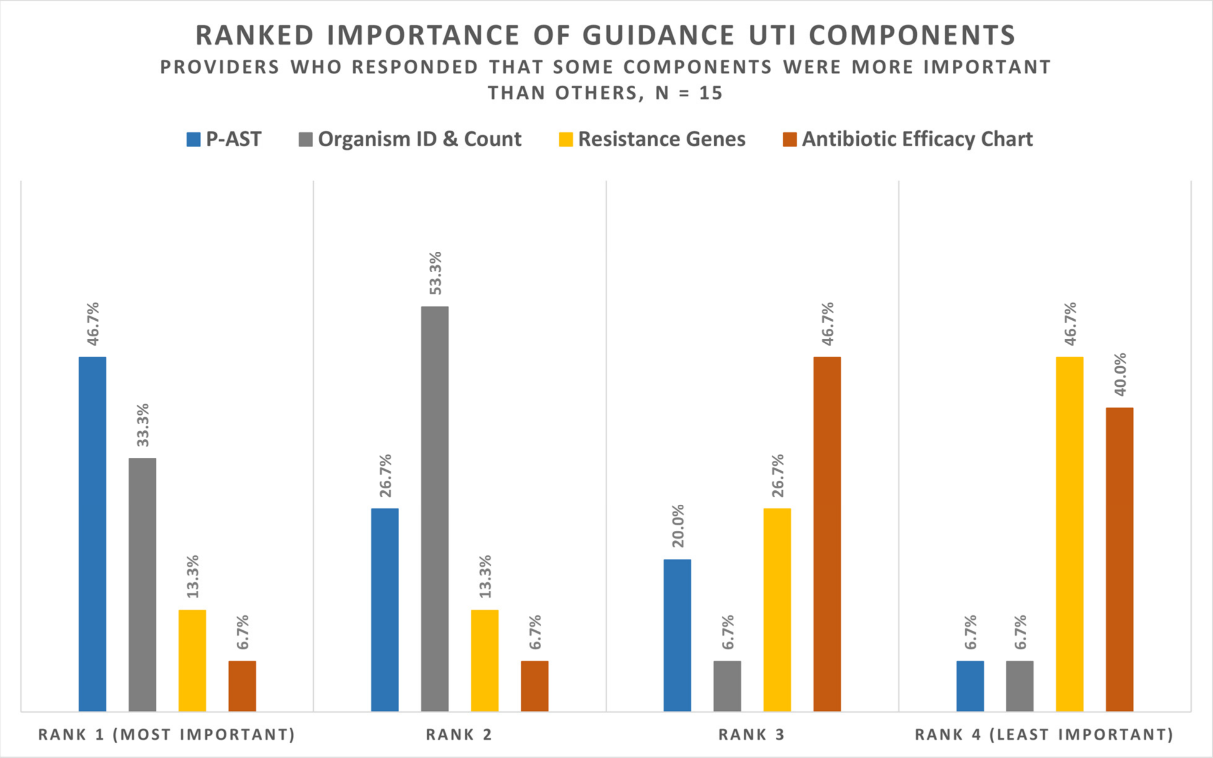

Supplement: Supplementary file 1 [file jcm-13-07453-s001.zip › jcm-3266537-supplementary.pdf]
